# Exploring rumen fermentation and microbial populations in Dhofari goats fed a chitosan-added diet

**DOI:** 10.1080/10495398.2024.2337748

**Published:** 2024-04-09

**Authors:** Hani M. El-Zaiat, Waleed Al-Marzooqi, Kaadhia Al-Kharousi

**Affiliations:** a Department of Animal and Veterinary Sciences, College of Agricultural and Marine Sciences, Sultan Qaboos University, Muscat, Sultanate of Oman; b Department of Animal and Fish Production, Faculty of Agriculture, Alexandria University, Alexandria, Egypt

**Keywords:** Chitosan additive, rumen fermentation, microbiome sequencing, Dhofari goats

## Abstract

The use of chitosan (CHI) in ruminant diets is a promising natural modifier for rumen fermentation, capable of modulating both the rumen pattern and microbial activities. The objective of this study was to explore the rumen fermentation and microbial populations in Dhofari goats fed a diet supplemented with CHI. A total of 24 Dhofari lactating goats (body weight, 27.32 ± 1.80 kg) were assigned randomly into three experimental groups (*n* = 8 ewes/group). Goats were fed a basal diet with either 0 (control), 180 (low), or 360 (high) mg CHI/kg of dietary dry matter (DM) for 45 days. Feeding high CHI linearly increased (*p* < 0.05) the propionate level and reduced the acetate, butyrate, and total protozoa count (*p* < 0.05). Ruminal ammonia nitrogen (NH_3_–N) concentrations and the acetate:propionate ratio decreased linearly when goats were fed CHI (*p* < 0.05). The abundances of both *Spirochetes* and *Fibrobacteres* phyla were reduced (*p* < 0.05) with both CHI doses relative to the control. Both low and high CHI reduced (*p* < 0.05) the relative abundances of *Butyrivibrio hungatei*, *Fibrobacter succinogenes*, *Ruminococcus albus*, *Ruminococcus flavefaciens*, *Selenomonas ruminantium* and *Neocallimastix californiae* populations. Adding CHI significantly decreased (*p* < 0.05) the abundances of *Ascomycota*, *Basidiomycota*, and *Bacillariophyta* phyla compared to the control. Adding CHI to the diet reduces the abundance of fibrolytic-degrading bacteria, however, it increases the amylolytic-degrading bacteria. Application of 360 mg of CHI/kg DM modified the relative populations of ruminal microbes, which could enhance the rumen fermentation patterns in Dhofari goats.

## Introduction

Ruminant adaptation to their environmental conditions was closely linked to their rumen microbial communities. Native breeds, like Dhofari goats, represent well-adapted genetic resources for their traditional production systems and unique local conditions. Understanding the rumen ecosystem and microbiome involved in feed digestion is essential for achieving more efficient feed conversion, thereby ensuring a sustainable supply of livestock products.^1^ The symbiotic relationship between the rumen microbiome and the host is crucial in providing dietary energy and essential nutrients, which ultimately influences animal health and productive performance.^2^ Furthermore, feeding patterns, feed additives, ration formulation, animal age and health conditions, and species, as well as their geographical origin, have been reported as factors affecting rumen microbiota composition and structure.^3–5^ Hence, introducing certain feed additives modulates ruminal fermentation responses and microbiome diversity, which, ultimately affects the host animal health and productivity.^6^ To achieve this, nutritionists are exploring natural alternatives to synthetic feed additives to alter the rumen ecosystem by modifying the balance and activity of the rumen microbiome, thereby promoting animal performance.^7^^,^^8^ Chitosan (CHI), a natural biopolymer produced by the deacetylation of chitin, has gained attention as a safe alternative to synthetic growth promoters in ruminant nutrition as a result of its antimicrobial properties against some rumen microbiota.^9^ However, reports concerning the efficacy of CHI provision on ruminal fermentation and the microbes community have not been extensively studied; few studies have reported conflicting results. Several studies have explored rumen microbial diversity using molecular-based techniques under particular feeding strategies.^10^^,^^11^ In an *in vitro* study, dietary addition with CHI shifted the fermentation pattern to greater acetate and less propionate, therefore minimizing the acetate-to-propionate ratio,^12^ representing a more energy-efficient pathway.^13^

The inclusion of CHI in the diet has shown antimicrobial potential, altering the rumen bacterial community structure and fermentation pattern to increase propionate production.^9^ Moreover, it has been reported that dietary administration with CHI decreases the relative abundances of ruminal cellulolytic (*Fibrobacter* and *Ruminococcus*) and hemicellulolytic-digesting bacteria (*Eubacterium* sp.).^14^ Furthermore, beef Heifers’ diet supplemented with CHI resulted in higher ruminal ammonia nitrogen (NH_3_–N) concentrations.^10^ To our knowledge, the present experiment is the first study focused on understanding and assessing the effect of CHI additives on the rumen microbiota and their fermentation metabolites in lactating Dhofari goats. We hypothesized that the antimicrobial capacity of CHI would modulate rumen microbial communities, favoring beneficial fermentation routes in lactating Dhofari goats. Therefore, the objective of this study was to investigate the ruminal fermentation attributes and microbial structures using molecular techniques in Dhofari goats fed a CHI-added diet.

## Materials and methods

All experimental protocols were reviewed and carried out based on the rules of both handling and care of animals approved by the Ethics Committee for Animal Use in Research of Sultan Qaboos University (Approval No. SQU/EC-AUR/2022-2023/5).

### Experimental animals, design, and diets

Twenty-four lactating Dhofari goats (27.32 ± 1.80 standard error (SE) kg of homogeneity in terms of average body weight) were randomly selected from goat flocks raised in the Agricultural Experiment Station, Sultan Qaboos University, Oman. The ewes were assigned to three experimental groups (*n* = 8 ewes/group). All ewes were housed in pens (approximately 1.7 × 0.7 × 1.5 m) equipped with accessible feeders, freshwater buckets, and mineral blocks throughout the experiment.

Goats were offered a basal diet (with 100 g/kg refusals) consisting of 400 g Rhodes grass/kg dry matter (DM) and 600 g concentrate feed mixture/kg DM (on a DM basis, Table 1) to meet the nutrient requirements according to NRC.^15^ Three diet treatments were assigned to three groups of ewes in a completely randomized design to receive either 0 (control), 180 (low), or 360 (high) mg CHI/kg of dietary DM. The CHI was ≥75% deacetylated, moisture 10%, and ash ≤2% (HIMEDIA Laboratories Pvt. Ltd., Mumbai, India). The dose of CHI was chosen based on the results from previous studies^12^^,^^14^^,^^16–18^ and a preliminary *in vitro* study (data not shown). The amount of CHI additive was weighed daily and manually administered to each ewe before the morning feeding to ensure that the ewes received a total amount of CHI. The concentrate was completely consumed by the animals within 15 min after delivery. The experiment lasted for 60 days, including a 15-day adaptation period. The experimental diet and CHI supplementation were offered once daily at 0900 h.

**Table 1.** Feed ingredient and composition of experimental diets (g/kg DM).

Item	Rhodes grass hay	Concentrate feed mixture	Experimental diet^a^
**Ingredients**			
Rhodes grass hay	–	–	400
Yellow corn	–	541	325
Wheat bran	–	206	124
Soybean meal	–	230	138
Sodium chloride	–	6.00	4.00
Limestone	–	14.0	8.00
Mineral mixture^b^	–	3.00	2.00
**Chemical composition**			
Organic matter	908	885	899
Extract ether	17.8	32.7	23.8
Crude protein	92.4	173	133
Neutral detergent fiber	715	325	559
Acid detergent fiber	486	108	335
Non-fiber carbohydrates^c^	197	443	183

^a^Consisted of 400 g Rhodes grass hay/kg DM and 600 g concentrate feed mixture /kg DM.

^b^Mineral and vitamin mixture provided the following: Ca, 1.00%; P, 0.60%; Se, 0.3 mg/kg; vit A, 8800 IU/kg; vit D, 2200 IU/kg and vit E, 33 IU/kg.

^c^Non-fiber carbohydrates (g/kg) = 1000 − (NDF + CP + EE + ash).

### Rumen fermentation parameters

Using a flexible tube made of polyvinylchloride (approximately 1 m in length); rumen sample contents (50 mL) were collected individually on days 15, 30, and 45 (approximately 2–3 h after feeding). Immediately after collection, rumen contents were squeezed, filtered, and rumen pH was measured (HANNA Instruments, HI Microcomputer 9025, pH Meter, Smithfield, VA, USA). Rumen content samples were transported to the Feed Analytical Laboratory located at Sultan Qaboos University within 20 min. At room temperature, a 2 mL subsample was combined with 4 mL of methyl green formalin saline solution in an Eppendorf tube (Eppendorf, 5702R, Hamburg, Germany), and subsequently preserved for total protozoa count using the Neubauer improved bright-line chamber (Labor Optik, Lancing, UK) following the procedure outlined by Dehority et asl.^19^ Three separate subsamples were centrifuged, and the supernatant was stored at −80 °C before the determination of ammonia nitrogen (NH_3_–N) concentration, short chain fatty acid (SCFA), and molecular analyses. The ruminal NH_3_–N concentration was measured by an automated COBAS® c111 biochemical analyzer (Roche Diagnostics International Ltd., Roche Rotkreuz, Switzerland) using commercial laboratory tests. The SCFA (acetate, propionate, iso-butyrate, butyrate, iso-valerate, and valerate) was measured using a gas chromatograph (GC, Agilent 6890 N) as described by Kholif et al.^20^ Aliquots of rumen subsamples were mixed with metaphosphoric acid (25%) and then centrifuged (Eppendorf, 5702 R, Hamburg, Germany) to separate the supernatant. In brief, the mixtures were centrifuged at 15,000×*g* for 20 min at 4 °C and immediately stored for subsequent SCFA analysis. A 10 µL mixture was injected into the GC to measure the individual SCFA concentrations based on their retention times, using a Volatile Acid Standard Mix (Supelco 46975-U, Bellefonte, PA, USA). The individual SCFA were separated using an HP-FFAP (Agilent 19091F-115) polyethylene glycol TPA capillary column (50 m × 0.320 mm i.d.) and detected with a flame ionization detector (FID) at a temperature of 260 °C. The carrier gas used was hydrogen with a flow rate of 40 mL/min. The EPC CIS3 inlet pressure was 12.78 psi, and the total flow was 78.3 mL/min. The analysis started at 80 °C for 1 min, increased to 120 °C for 3 min, followed by a 6.03 min ramp to 205 °C, and then held for 3 min, with a final detector temperature of 240 °C.

### Deoxyribonucleic acid (DNA) extraction, sequencing, and bioinformatics analysis

Ruminal content samples were thawed at 4 °C for 24 h and then homogenized for 1 min in a blender. Microbial genomic DNA from each rumen content (containing both solid and liquid fractions) was extracted using the QIAamp DNA Stool Mini Kit (Qiagen, CA, Hamburg, Germany), according to the manufacturer’s guidelines. The DNA concentration was then assessed using NanoDrop 2000 (Thermo Electron Corporation, Waltham, MA, USA) by measuring the optical density at wavelengths of 260 and 280 nm. The quality of the extracted DNA was determined using a NanoDrop ND-1000 spectrophotometer (Nyxor Biotech, Paris, France).

For bacterial community analysis, the hypervariable regions V3–V4 of the bacterial 16S rRNA gene were selected for polymerase chain reaction (PCR) amplification using the universal primers (338F: 5′-ACTCCTACGGGAGGCAGCAG-3′) and (806 R: 5′ GGACTACHVGGGTWTCTAAT-3′).^21^ For fungal community sequences, extracted DNA was used for PCR amplification of the internal transcribed spacer (ITS) primer (ITS1: 5′‑GGAAGTAAAAGTCGTAACAAGG‑3′) and ITS2: 5′‑GCTGCGTTCTTCATCGATGC‑3)^22^ as the targeted region for exploring the diversity of rumen fungi (18S rRNA gene). The PCR conditions were performed using the protocol described by Cui et al. ^23^ Briefly, PCR programs were carried out in triplicate (16S rRNA and 18S rRNA genes) with a 50 μL reaction mixture comprising 30 ng of qualified DNA template, 4 μL of PCR primer cocktail (16S V3-V4); 18S amplify the ITS1 and ITS2 regions; the primers ITS1-ITS2 are for the ITS1 region and primer ITS3–ITS4 for the ITS2 region; and 25 μL of PCR Master Mix (NEB Phusion High-Fidelity PCR Master Mix). The PCR conditions were as follows: 3 min of initial denaturation at 98 °C followed by 30 cycles, annealing at 55 °C for 45 s, elongation at 72 °C for 45 s, and then 7 min of final extension at 72 °C. All PCR-amplified products were purified using Agencourt AmpureXPbeads (AGENCOURT), dissolved in the elution buffer, and labeled to complete library construction. Library size and concentration were identified by using an Agilent 2100 Bioanalyzer instrument (Agilent DNA 1000, Agilent Technologies, China). The qualified libraries were sequenced based on their insert sizes by the BGI Genomic Lab, Tai Po Industrial Zone (New Territories, Hong Kong, China). Uparse Software (V.7.0.1090) was used to cluster the sequence reads into operational taxonomic units (OTUs) with a 97% similarity. However, chimeras in OUTs were compared with the gold database using Uchime (v4.2.40). Then, OTU representative sequences were classified using the Ribosomal Database Project Classifier v.2.2 with a minimum confidence threshold of 0.6 and trained on the Greengenes database v201305 by Qiime (v1.8.0). The Usearch-global was used to compare all tags back to OTU to get the OTU abundance statistics table for each sample. The alpha diversity attributes (including observed species, Chao, Ace, Shannon, Simpson, and good-coverage indices) of microbial communities in each treatment were estimated by Mothur (v.1.31.2) and Qiime (v1.8.0) at the OTU level, respectively. A sample cluster was conducted by Qiime (v1.8.0) based on Upgma. A barplot of different classification levels was plotted with the R package v3.4.1. Principal Coordinate Analysis (PCoA) at the OUT level was conducted by Qiime (v1.8.0) to estimate the similarity or dissimilarity of data among treatments. The Venn plots in OTUs were plotted using the R package “VennDiagram” (version 3.1.1.).

### Laboratory analysis of feed

Samples of feed were collected weekly and stored at −80 °C until analysis. All samples were thawed, dried at 60 °C for 48 h and then ground through a 1-mm screen using a mill (Tector; CEMOTEC, 1090 mill, Sweden) for subsequent chemical analysis. According to AOAC,^24^ samples were analyzed for dry matter (DM) and organic matter (OM) contents. Crude protein (CP) was determined using a nitrogen analyzer (BUCHI KjelMaster K-375, Flawil, Switzerland). Ether extract (EE) was determined using petroleum ether by Soxhlet extraction of the dry sample (ID method 920.39). Neutral detergent fiber (NDF) was determined as described by Van Soest et al.^25^ however, acid detergent fiber (ADF) was determined by using the methodology described by Roberston and Van Soest.^26^ Non-fiber carbohydrates (NFC, g/kg) were calculated as 1000 − (NDF + CP + EE + Ash).

### Statistical analysis

The data of ruminal fermentation metabolites were analyzed using the general linear model (GLM) procedure of SAS (v.9.1, SAS 2002 Inst. Inc., Cary, NC, USA) according to the following statistical model: *Y_ij_* = *μ* + *T_i_* + *E_ij_*_,_ where *Y_ij_* = observations mean, *μ* = general mean, *T_i_* = the fixed effect of experimental treatments (*i* = control, low, and/or high), and *E_ij_* = the experimental error. Ruminal fermentation constituents were subjected to analysis of variance (ANOVA), and linear and quadratic polynomial contrasts were applied to assess the effect of the CHI dose on the measured parameters. Statistical differences between treatments were defined at *p* ≤ 0.05, whereas the tendency of the difference was declared at 0.05 < *p* ≤ 0.10. The relative abundances of microbial communities were compared among sampling groups using non-parametric tests (Kruskal-Wallis test) and ANOVA analysis. The alpha diversity of the rumen microbiota data was tested using the Kruskal-Wallis test to evaluate differences among the three treatment groups. Analysis of similarities (ANOSIM) was used to analyze PCoA data.

## Results

### Ruminal fermentation constituents

No significant difference in the mean values of ruminal pH and total SCFA with the dietary addition of CHI was observed relative to the control diet (Table 2). Ruminal NH_3_–N concentration was reduced (*p* < 0.05) by 24% in goats fed both low and high treatments compared to those fed the control (average 16.80 vs. 22.18, respectively). Moreover, goats fed a high CHI diet reduced acetate and butyrate (*p* < 0.05), but increased propionate (*p* < 0.05) in comparison to the control. Provision of both low and high CHI-added diets showed a decreased (*p* < 0.05) acetate-to-propionate ratio compared to the control group. Compared to the low CHI group, ruminal butyrate, and acetate-to-propionate ratios were lower (*p* < 0.05) in the high CHI additive group. Compared with both the control and low groups, a diet supplemented with highs showed a significant reduction (*p* < 0.05) in total protozoa count. Feeding high CHI linearly increased (*p* < 0.05) the propionate level and reduced the acetate, butyrate, and total protozoa count (*p* < 0.05). The ruminal ammonia nitrogen concentration and acetate: propionate ratio had a linear decrease when goats were fed CHI (*p* < 0.05).

**Table 2.** Effect of chitosan (CHI) supplementation on rumen fermentation parameters of Dhofari goat.

Item	Chitosan dose mg/kg DM of concentrate			SEM	*p*-value		
	Control	Low	High		Treatment	Linear	Quadratic
pH	6.66	6.65	6.70	0.04	0.785	0.993	0.812
NH_3_-N, mg/dL	22.18^a^	17.30^b^	16.29^b^	0.66	<0.001	0.016	0.189
Short-chain fatty acids, mmol/L							
Total	100.12	97.96	96.99	1.38	0.281	0.283	0.434
Acetate	71.97^a^	68.58^ab^	66.85^b^	0.95	0.020	0.038	0.203
Propionate	16.33^c^	18.63^b^	20.89^a^	0.45	<0.001	0.004	0.382
Iso-butyrate	0.82	0.80	0.83	0.03	0.806	0.547	0.432
Butyrate	9.02^a^	8.04^b^	7.25^c^	0.17	<0.001	0.006	0.991
Iso-valerate	0.99	0.99	1.03	0.03	0.626	0.292	0.369
Valerate	1.03	1.23	1.21	0.09	0.268	0.114	0.205
Acetate to propionate ratio	4.42^a^	3.70^b^	3.22^c^	0.12	<0.001	<0.001	0.109
Protozoa count, ×10^5^ cell/mL	3.13^a^	2.65^b^	1.53^c^	0.19	<0.001	0.047	0.553

Control: basal diet without CHI; low: basal diet plus 300 mg CHI/kg DM of concentrate; high: basal diet plus 600 mg CHI/kg DM of concentrate.

NH_3_-N: ammonia nitrogen; SEM: standard error of the mean.

Mean values within a row with unlike superscript letters were significantly different (*p* < 0.05).

### Microbial community analyses

**Figure 1.**
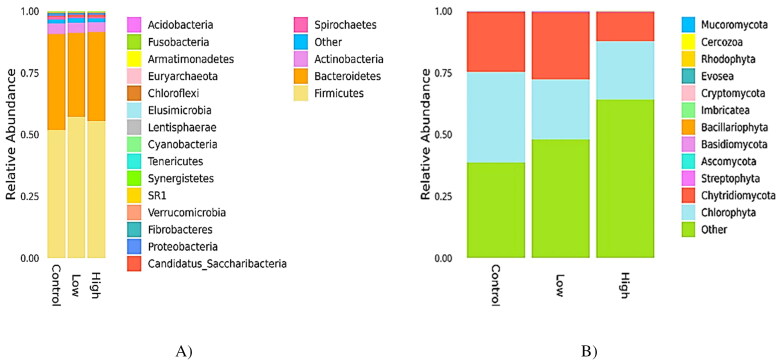
Effect of chitosan (CHI) supplementation on relative abundances of rumen bacterial and fungal communities classified at phylum-level of Dhofari goat (*n* = 27 per treatment); control: basal diet without CHI; low: basal diet plus 300 mg CHI/kg DM of concentrate; high: basal diet plus 600 mg CHI/kg DM of concentrate. (A) Bacterial communities. (B) Fungal communities.

The relative abundances of the bacterial community were considerably higher than those of the fungal community. The relative abundances (in percentage) of rumen bacterial communities at the phylum and species level in goats fed a CHI-added diet are presented in Table 3 and Figure 1. At the phylum level, Firmicutes (54.33%), Bacteroidetes (37.59%), and Actinobacteria (2.94%) were predominant in all samples (Figure 1A). No significant differences in the abundance of Firmicutes and Bacteroidetes populations were observed among the three groups (*p* > 0.05). Both low and high goats fed a CHI-added diet reduced the relative abundances of Spirochetes and Fibrobacteres (*p* < 0.05) compared to the control. The relative abundance of *Candidatus Saccharibacteria* was increased (*p* < 0.05) in ewes fed with a high CHI-added diet in comparison to those fed with low CHI and control diets. At the species level, 156 species were quantified, and the most abundant species were clarified in all the samples (Table 3 and Figure 1). Goats fed both CHI-added diets resulted in a higher (*p* < 0.05) TM7 phyla, *Barnesiella intestinihominis*, and *Eubacterium sulci* taxa relative to those fed the control diet. Among the cellulolytic bacteria determined, *Butyrivibrio hungatei*, *Fibrobacter succinogenes*, *Ruminococcus albus*, *Ruminococcus flavefaciens*, and *Selenomonas ruminantium* were significantly decreased (*p* < 0.05) with the addition of CHI compared with the control. In addition, the relative abundance of Prevotella baroniae was reduced (*p* < 0.05) in goats supplemented with low than in those fed with high or a control diet.

**Table 3.** Effect of chitosan (CHI) supplementation on relative abundances (in percentage) of the most abundant rumen bacterial 16S rRNA of Dhofari goat at phylum and species level as determined by the Kruskal’s test (*n* = 27 per treatment).

Taxon	Chitosan dose mg/kg DM of concentrate			SEM	*p*-value
	Control	Low	High		
**Phylum**					
* Firmicutes*	51.58	55.04	56.39	0.390	0.237
* Bacteroidetes*	38.71	37.70	36.37	0.184	0.384
* Actinobacteria*	3.27	2.39	3.15	0.074	0.797
* Spirochaetes*	1.01^a^	0.52^b^	0.63^b^	0.040	0.007
* Candidatus_Saccharibacteria*	0.51^b^	0.72^ab^	0.79^a^	0.023	0.050
* Proteobacteria*	0.54	0.52	0.53	0.002	0.708
* Fibrobacteres*	0.53^a^	0.23^b^	0.32^b^	0.024	0.001
* Verrucomicrobia*	0.21	0.22	0.24	0.002	0.814
* SR1*	0.18	0.26	0.20	0.007	0.758
* Tenericutes*	0.08	0.11	0.07	0.003	0.107
**Species**					
* Prevotella brevis*	5.15	4.23	4.18	0.086	0.758
* Prevotella oralis*	0.93	0.65	1.14	0.019	0.459
* Ruminococcus bromii*	2.19	2.39	2.94	0.067	0.249
* Saccharofermentans acetigenes*	1.54	1.86	1.63	0.008	0.564
* Succiniclasticum ruminis*	1.26	1.19	0.77	0.044	0.459
* TM7_phylum*	0.51^b^	0.72^ab^	0.79^a^	0.025	0.049
* Eubacterium coprostanoligenes*	0.54	0.61	0.65	0.009	0.110
* Barnesiella intestinihominis*	0.27^b^	0.64^a^	0.55^a^	0.026	0.001
* Butyrivibrio hungatei*	0.68^a^	0.36^b^	0.35^b^	0.030	0.020
* Fibrobacter succinogenes*	0.53^a^	0.22^b^	0.32^b^	0.019	0.001
* Ruminococcus albus*	0.25^a^	0.19^b^	0.17^b^	0.007	0.049
* Ruminococcus flavefaciens*	0.22^a^	0.13^b^	0.12^b^	0.009	0.027
* Selenomonas ruminantium*	0.07^a^	0.04^b^	0.05^ab^	0.003	0.020
* Eubacterium sulci*	0.03^b^	0.05^a^	0.06^a^	0.002	0.039
* Methanobrevibacter millerae*	0.007	0.004	0.003	0.003	0.159
* Prevotella baroniae*	0.20^a^	0.02^b^	0.25^a^	0.019	0.003

Control: basal diet without CHI; low: basal diet plus 300 mg CHI/kg DM of concentrate; high: basal diet plus 600 mg CHI/kg DM of concentrate; SEM: standard error of the mean; mean values within a row with unlike *superscript letters* were significantly different (*p* < 0.05).

The relative abundance (in percentage) of the rumen fungal community at the phylum and species level is shown in Table 4 and Figure 1. Besides the phylum, the fungal community species were also identified. In all samples, Chlorophyta and Chytridiomycota were identified as the predominant phyla, which represented 25.26 and 18.75% of the fungal community, respectively (Figure 1B). Adding CHI to the diet significantly decreased the taxa of Ascomycota, Basidiomycota, and Bacillariophyta (*p* < 0.05) phyla relative to the control. Compared to the control group, the addition of CHI to the diet decreased (*p* < 0.05) the relative abundance of *Neocallimastix californiae* species (Table 4).

**Table 4.** Effect of chitosan (CHI) supplementation on relative abundances (in percentage) of the most abundant rumen fungal 18S rRNA communities of Dhofari goat at phylum and species level as determined by the Kruskal’s test (*n* = 27 per treatment).

Taxon	Chitosan dose mg/kg DM of concentrate			SEM	*p*-value
	Control	Low	High		
**Phylum**					
* Chlorophyta*	32.491	18.912	24.395	1.073	0.237
* Chytridiomycota*	23.016	23.461	9.780	1.221	0.384
* Streptophyta*	0.096	0.141	0.112	0.004	0.797
* Ascomycota*	0.135^a^	0.077^b^	0.089^b^	0.005	0.007
* Basidiomycota*	0.054^a^	0.024^b^	0.024^b^	0.003	0.049
* Imbricatea*	0.012	0.008	0.008	0.000	0.708
* Bacillariophyta*	0.014^a^	0.000^b^	0.012^ab^	0.001	0.001
**Species**					
* Pecoramyces ruminatium*	11.13	12.83	4.36	0.704	0.118
* Fungal_sp_Tianzhu_Yak19*	5.19	10.60	8.42	0.428	0.869
* Neocallimastix californiae*	1.77^a^	1.76^a^	0.47^b^	0.117	0.011
* Bysmatrum arenicola*	0.28	0.29	0.20	0.008	0.849
* Corchorus olitorius*	0.10	0.14	0.11	0.004	0.898
* Pinnularia appendiculata*	0.012	0.001	0.014	0.001	0.413
* Claviceps purpurea*	0.002	0.002	0.008	0.001	0.468

Control: basal diet without CHI; low: basal diet plus 300 mg CHI/kg DM of concentrate; high: basal diet plus 600 mg CHI/kg DM of concentrate; SEM: Standard error of the mean; mean values within a row with unlike superscript letters were significantly different (*p* < 0.05).

**Figure 2.**
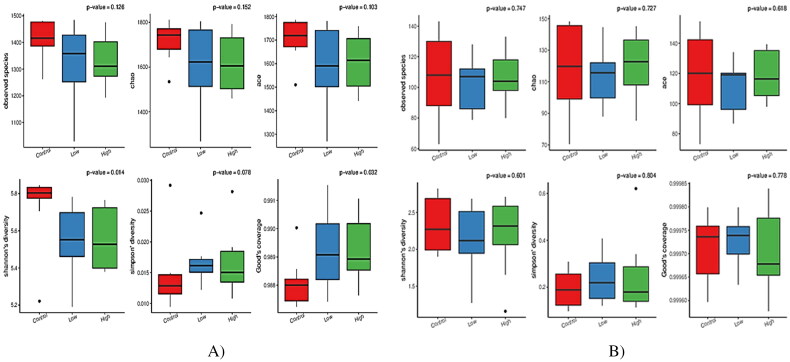
Comparison of the alpha-diversity parameters of rumen bacterial and fungal communities of Dhofari goat between treatments (*n* = 27 per treatment); control: basal diet without CHI; low: basal diet plus 300 mg CHI/kg DM of concentrate; high: basal diet plus 600 mg CHI/kg DM of concentrate. The significantly different bacterial and protozoal communities between treatments are indicated by *p* < 0.05. (A) Bacterial communities. (B) Fungal communities

**Figure 3.**
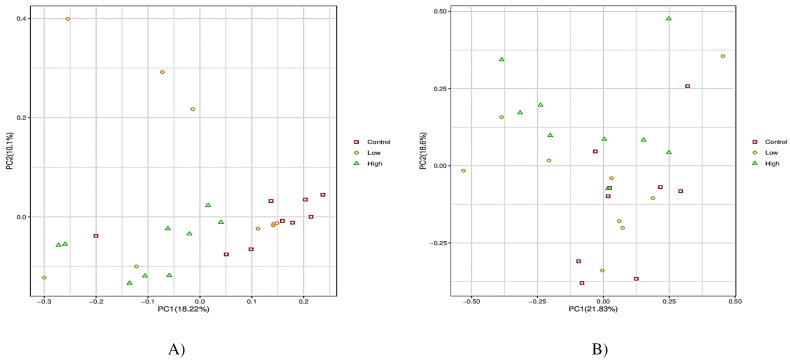
Principal coordinate analysis (PCoA) plots on OTU level of OTUs of rumen bacterial and fungal community of Dhofari goat between the three treatments (*n* = 27 per treatment) using an unweighted UniFrac metric: Control: basal diet without CHI; low: basal diet plus 300 mg CHI/kg DM of concentrate; high: basal diet plus 600 mg CHI/kg DM of concentrate. (A) Bacterial communities. (B) Fungal communities.

**Figure 4.**
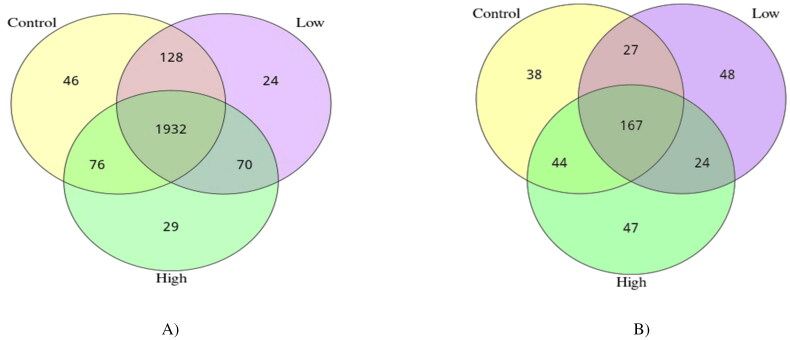
Venn diagram of OTUs of rumen bacterial 16S rRNA (specific to the V3–V4 region) and fungal 18S rRNA (specific to the V4 region) of Dhofari goat between treatments (*n* = 27 per treatment); control: basal diet without CHI; low: basal diet plus 300 mg CHI/kg DM of concentrate; high: basal diet plus 600 mg CHI/kg DM of concentrate. (A) Bacterial communities. (B) Fungal communities.

The alpha-diversity indices were used to analyze the diversity of bacterial and fungal populations in the rumen (Table 5 and Figure 2). Both low and high groups reduced (*p* < 0.05) the Shannon index and increased (*p* < 0.05) the good coverage compared with the control group. In the case of the fungal community, no groups displayed significant differences in alpha diversity indices (Figure 2B). However, PCoA discriminated in response to CHI additives based on supplement level (Figure 3). For the Rumen fungal community, the first two principal components (PC1 and PC2) accounted for 21.83 and 18.60% of the total variation among samples, respectively. In all treatments, no distinct clustering patterns for the rumen bacterial and fungal communities based on the CHI supplement were observed along the PCoA (Figure 3A and B). A total of 2305 OTUs were generated from 24 goats by gene sequencing, of which 1932 coexisted among all treatment groups and were considered core bacterial OTUs (the core OTUs represent 83.8% of the total OUTs, Figure 4A). For rumen fungal communities, a total of 128 ruminal bacteria OTUs were shared between the control and low groups, 70 OTUs between low and high treatment, and 76 OTUs between high and control (Figure 4A). The taxonomic analysis reflected a total of 395 OTUs from the 24 samples, corresponding to three treatments. Venn diagrams revealed the core set of 167 fungal OTUs shared across the three treatment groups (Figure 4B). Furthermore, there were 38, 48, and 47 OTUs that were exclusively identified in the control, low, and high groups, respectively (Figure 4B).

**Table 5.** Effect of chitosan (CHI) supplementation on alpha-diversity indices of rumen bacterial and fungal communities of Dhofari goat (*n* = 27 per treatment).

Indices	Chitosan dose mg/kg DM of concentrate			SEM	*p*-value
	Control	Low	High		
**Bacterial communities**					
* Observed species*	1417	1330	1327	19.861	0.126
* Chao*	1717	1610	1617	24.259	0.152
* Ace*	1704	1598	1606	23.206	0.103
* Shannon*	5.74^a^	5.54^b^	5.56^b^	0.037	0.014
* Simpson*	0.014	0.017	0.016	0.001	0.078
* Good’s coverage*	0.988^b^	0.989^a^	0.989^a^	0.000	0.032
**Fungal communities**					
* Observed species*	105.9	101.4	107.8	3.865	0.747
* Chao*	116.6	112.7	119.9	4.211	0.727
* Ace*	119.4	113.2	119.6	3.904	0.618
* Shannon*	2.34	2.17	2.21	0.086	0.601
* Simpson*	0.198	0.232	0.239	0.022	0.804
* Good’s coverage*	1.00	1.00	1.00	0.000	0.778

Control: basal diet without CHI; low: basal diet plus 300 mg CHI/kg DM of concentrate; high: basal diet plus 600 mg CHI/kg DM of concentrate; SEM: standard error of the mean; mean values within the same row with different superscript letters were significantly different (*p* < 0.05).

## Discussion

In the current study, regardless of the provision of CHI additives in the diet, ruminal pH values remained close to neutrality (average 6.67), indicating that the tested CHI doses have similar potential on ruminal pH.^12^ However, CHI molecules exhibit polyatomic properties when the pH falls below their pKa, which typically occurs within the range of 6.3 to 6.5.^27^ The decrease in ruminal NH_3_-N concentration suggests a possibility of reduced protein degradation in the rumen and increased CP digestibility,^28^ in response to CHI provision. The CHI effects on ruminal NH_3_-N concentration in the present study are paralleled with the observation of Goiri et al.^29^ who pointed out the reduction in NH_3_–N concentration with the inclusion of CHI in the sheep diet. Under such ruminal pH conditions, the interaction of the positive charge of amine groups in CHI with the negative charge of carboxyl groups in amino acids causes lower protein degradation in the rumen, which could explain the lower NH_3_–N concentrations.^30^ In contrast, the opposite concentrations of ruminal NH_3_–N were reported in a previous study.^13^ Dietary and microbial protein degradation are positively associated with increased protozoa activity in the rumen, resulting in greater production of ruminal NH_3_–N. Therefore, the decreased concentration of ruminal NH_3_–N observed in the present study can be attributed to the reduction in total protozoa abundance. Besides, CHI addition significantly reduced the total protozoa number in the rumen through the strong antiprotozoal potential of CHI additive.^31^ The decreased ruminal NH_3_-N rates may negatively affect the proliferation of the fibrinolytic bacteria community,^32^ which is consistent with our findings and thus leads to lower acetate production.

Dietary addition with CHI potentially suppresses the growth and activities of gram-positive bacteria such as *Ruminococcus albus*, *Ruminococcus flavefaciens*, and *Selenomonas ruminantium* observed in our study. This suppression could explain the lower acetate production relative to the control. These findings may be a consequence of the effect of CHI provision, which shifted the bacterial community toward more propionate and less acetate, resulting in a declining acetate-to-propionate ratio^30^ without altering total SCFA in the rumen.^12^^,^^13^^,^^30^ The observed reduction in the acetate-to-propionate ratio is directly linked to CHI’s negative impact on the relative abundances of ruminal *Ruminococcaceae* (as a cellulolytic gram-positive bacteria), leading to changes in the bacterial microbiome structure and their fermentation end-products.^31^ Therefore, the decrease in the abundance of *Fibrobacter succinogenes* in CHI-supplemented goats further confirmed the adverse potential of CHI on fibrinolytic bacteria. In the current study, a decrease in ruminal acetate is expected in goats fed CHI-added diets because of the decrease in relative abundances of ruminal *Spirochetes* and *Fibrobacteres*. The reduction in the relative abundances of rumen fibrinolytic bacteria (such as *Fibrobacter*, *Butyrivibrio*, and *Ruminococcus*) in CHI-added diets was found to be closely associated with their rumen fermentation end-products, which is consistent with the results reported by Belanche et al.^14^

The bioinformatics analysis revealed that *Firmicutes* and *Bacteroidetes* were the core microbiome in the rumen, regardless of the animal species, accounting for about 91.9% of the total abundance. This finding indicates that these phyla play a crucial role in the rumen functions of ruminants, revealing the relationship between such ruminal microbes and their fermentation metabolites. Thus, we speculated that CHI additives might diminish fiber degradability as a result of a decreased relative abundance of *Spirochetes* and *Fibrobacteres*. Golder et al.^33^ reported that *Spirochetes* and *Fibrobacteres* are positively associated with fiber degradation, which supports the finding observed in our study. In the present study, a noticeable increase in the relative abundance of TM7^33^ was observed in response to CHI addition. The amylolytic fermentative pattern of TM7 may have contributed to the greater propionate production. This could be based on the presence of TM7 was mainly linked to lactate production,^34^ suggesting a possibility of higher propionate levels observed in our study. This typically indicates a greater rate of dietary energy utilization during fermentation, resulting in greater energy available for metabolism. The relative abundances of *Barnesiella intestinihominis* (a Gram-negative bacteria) increased, suggesting that CHI provision could promote the growth of these bacteria. Furthermore, *Barnesiella intestinihominis* is a well-known saccharolytic bacteria that produces succinate.^35^ Therefore, the greater propionate observed in goats fed CHI-added diets may be attributed to the succinate produced by these bacteria.

The antimicrobial action of CHI is attributed to both hydrophobic and chelating potentials when the environmental pH exceeds the pKa of CHI.^36^ Another possible explanation for such ruminal changes is the inhibitory action of CHI against gram-positive bacteria rather than gram-negative bacteria.^9^^,^^18^ Furthermore, the antimicrobial effects of CHI on altering microbial populations and consequently their final products in the rumen are mostly similar to changes observed when ionophores were supplemented.^9^ The antimicrobial mechanism of CHI is reasonably associated with its effectiveness in altering cell membrane permeability due to the interactions between CHI's polycationic nature and the negative charges on the outer cell membrane of bacteria.^18^ Notably, goats fed a high CHI-added diet showed the lowest values of butyrate, while low treatment had an intermediate value. The negative correlation between TM7 species and butyrate might indicate that these bacteria can compete with butyrate-producing species for the same substrate or use butyrate for their metabolic purposes. In our study, the reduction in ruminal butyrate was positively correlated with the decreased relative abundance of *Butyrivibrio* in response to CHI addition. This finding could be due to the capability of *Butyrivibrio* spp. to use complex structural carbohydrates to produce butyrate.^37^ Taken together, these changes in fermentation parameters imply an alteration in the microbial population in the rumen. However, the current study revealed limited differences in the effect of CHI on rumen microbial communities, which is partly in line with previous publications.^9^^,^^18^ In this study, the results of 16S and 18S rRNA gene sequencing revealed clear evidence of CHI’s antimicrobial potential against several ruminal bacteria and fungi.

In the current study, the abundance of *Prevotella brevis* was not affected by CHI inclusion in the diet, but the number of *Prevotella brevis* tended to be reduced in the goats fed CHI-added diets compared to the control. Moreover, the relative abundance of the *Prevotella baroniae* population was positively accompanied by propionate production in the high treatment group, highlighting the potential of such microbes on the changes in the rumen fermentation pattern. The difference observed in the abundance of *Prevotella* spp. likely explains that the mode of action of CHI is strain-independent within *Prevotella* species. The ruminal *Prevotella* spp. has been associated with producing succinate (a propionate precursor) due to its ability to utilize feed carbohydrates and proteins, which are further converted to propionate.^38^ This is consistent with previous reports describing the potential of CHI as a feed additive in ruminant diets based on its antimicrobial properties.^36^

The current study is the first to display original information on the diversity of rumen fungal communities in Dhofari goats. Previous studies have pointed out that the *Ascomycota* and *Basidiomycota* phyla have a crucial role in ruminal fiber degradability of goats.^39^ Notably, although the differences in diversity of rumen fungal at level species were insignificant, however, the abundance of *Neocallimastix californiae* decreased in response to high treatment. The *Neocallimastix sp.* is considered one of the most predominant fiber-degrading fungi in the rumen microbiome.^40^ Here, we can hypothesize that the significant changes in acetate and propionate proportions are likely associated with the reduction in *Neocallimastix californiae*. This finding indicates that the depressive ability of CHI on the rumen would negatively affect the proliferation of fungal communities involved in fiber degradation in the rumen.

In terms of the alpha diversity of rumen bacterial communities, CHI addition did not affect the Chao index, indicating similar sequencing depth among all treatments. Moreover, CHI-added diets reduced the Shannon index, indicating a decrease in bacterial diversity and activity in the rumen. However, the Good’s coverage was greater and more than 98% in all groups, representing improved sampling and sequencing accuracy across all species. However, no differences in terms of alpha diversity among all treatments for fungal populations were observed. Compared to ruminal bacteria, our results demonstrate that fungal communities seemed to be less susceptible to the provision of CHI. The PCoA revealed that the bacterial community diversity in the rumen accounted for 18.22% of the variation with high treatment, relative to both the low and control groups by PC1, while the difference between the low treatment and the control diet explained 10.10% of the variation in PC2. For the fungal community structure, the PC1 explained 21.83% of the fungal community diversity, while the PC2 only explained 18.6%. Such inconsistent results are partly related to the variations in physicochemical characteristics (such as degree of deacetylation and molecular weight) of CHI, dose usage, or diet composition.^16^^,^^30^^,^^41^

## Conclusion

The inclusion of CHI shifted the rumen fermentation attributes toward less acetate and greater propionate while reducing the total protozoa abundance without affecting pH or total SCFA. The provision of CHI in the diet reduces the relative abundance of fibrinolytic-degrading (*Ruminococcus albus*, *Ruminococcus flavefaciens*, *Selenomonas ruminantium*, *Fibrobacter succinogenes*, *Butyrivibrio*) bacteria but increases the amylolytic-degrading (*TM7* phylum, *Prevotella* species) bacteria. Furthermore, adding 360 mg CHI/kg of dietary DM demonstrated a significant effect on bacterial populations and structure in Dhofari goats under traditional feeding conditions. Our data represents preliminary scientific evidence for developing nutritional interventions to enhance the performance and productivity of Dhofari goats.
